# Soluble molecule profiling and network analysis of primary Sjögren's Syndrome patient serum

**DOI:** 10.1186/1471-2474-14-S1-A2

**Published:** 2013-02-14

**Authors:** JR Tarn, A Natasari, S Mitchell, S Bowman, E Price, C Pease, P Emery, J Andrews, M Bombardieri, N Sutcliffe, C Pitzalis, P Lanyon, J McLaren, J Hunter, M Gupta, M Regan, A Cooper, I Giles, D Isenberg, D Young, D Lendrem, C Downie, H Foggo, P Stocks, S Edgar, WF Ng

**Affiliations:** 1 Musculoskeletal Research Group, Institute of Cellular Medicine, Newcastle University, UK; 2 University Hospital Birmingham, Birmingham, UK; 3 Great Western Hospitals NHS Foundation Trust, Swindon, UK; 4 Section of Musculoskeletal Disease, Leeds Institute of Molecular Medicine, University of Leeds & NIHR Leeds Musculoskeletal Biomedical Research Unit, Leeds Teaching Hospitals Trust, Leeds, UK; 5 Barts and the London NHS Trust, UK; 6 Nottingham University Hospital, Nottingham, UK; 7 NHS Fife, Whyteman’s Brae Hospital, Kirkcaldy, UK; 8 Gartnavel General Hospital, Glasgow, UK; 9 Royal Derby Hospital, Derby, UK; 10 Royal Hampshire County Hospital, Winchester, UK; 11 University College London Hospitals NHS Foundation Trust, London, UK

## Background

Primary Sjögren’s Syndrome (pSS) is a chronic autoimmune syndrome characterised by sicca symptoms, fatigue, musculoskeletal pain and an increased risk of lymphoma. Patient populations are notably heterogeneous in their symptoms, adding to the challenge of pSS research. This study utilises serum samples from the UK Primary Sjögren’s Syndrome Registry (UKPSSR) - a large cohort of clinically well-characterised pSS patients and healthy controls with an aim to determine whether serum cytokines, chemokines and adhesion molecules may be used to differentiate pSS patients from healthy controls.

## Methods

Serum levels of 24 different analytes for 150 pSS patients and 30 age matched healthy controls were measured using Cytometric Bead Array (BD Biosciences).

The primary Sjögren’s Syndrome subjects (characterised by AECG criteria) were stratified as follows:

• Lymphoma and/or paraprotein positive;

• High systemic disease activity (ESSDAI score > 12);

• High Fatigue (VAS score >85);

• Low residual glandular function (OSF <1 ml/15 min and Schirmer’s test <1 cm)

The relationship between the levels of each analyte and clinical and laboratory parameters of PSS was examined using multivariate analysis and Mann-Whitney U testing; p-values were adjusted for multiple comparisons using Bonferroni’s correction.

## Results

There were marked differences in the levels of 11 analytes between pSS patients and healthy controls, with a p value <0.001, statistically significant after Bonferroni’s correction for multiple comparisons. However, none of the serum factors measured significantly differentiate different Sjögren’s subsets after multiple comparison correction.

**Figure 1:**
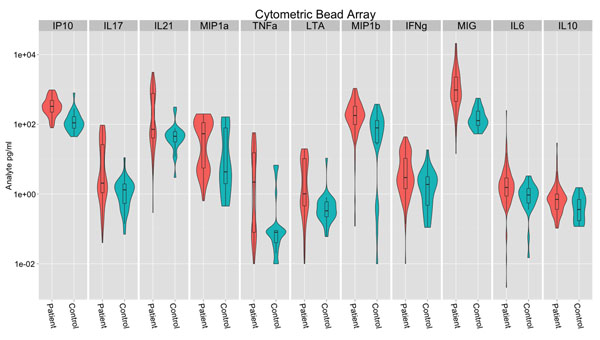


The violin-box plots in Figure 1 show the data distribution. Most soluble molecules show an ‘hour-glass’ distribution, particularly within the patient group corresponding to groups of patients with higher and lower levels of analyte.

## Discussion

Differences in blood cytokine and chemokine levels between primary Sjögren’s Syndrome patients and controls can be detected in serum through the use of Cytometric Bead Arrays. 11 serum analytes differ significantly between patients and controls: IP10 (CXCL10), IL17, IL21, IFNa, MIP1a (CCL3), LTA and TNFa, MIP1b (CCL4), IFNa, MIG (CXCL9), IL6 and IL10. Our observations raise the possibility that these analytes may be important in disease pathogenesis.

